# Key Considerations for Policymakers—Iodized Salt as a Vehicle for Iron Fortification: Current Evidence, Challenges, and Knowledge Gaps

**DOI:** 10.1093/jn/nxaa377

**Published:** 2021-02-15

**Authors:** Adam Drewnowski, Greg S Garrett, Rishi Kansagra, Noor Khan, Roland Kupka, Anura V Kurpad, Venkatesh Mannar, Reynaldo Martorell, Michael B Zimmermann, for the Double Fortified Salt Consultation (DFS) Steering Group, Omar Dary, Rafael Flores-Ayala, Dipika Matthias

**Affiliations:** Center for Public Health Nutrition, University of Washington, Seattle, WA, USA; ThinkWell, Geneva, Switzerland; Global Alliance for Improved Nutrition (GAIN), Geneva, Switzerland; PureBond Ltd, London, United Kingdom; Nutrition International, Ottawa, Canada; United Nations Children's Fund (UNICEF) Headquarters, New York, NY, USA; Department of Physiology, St. John's Medical College, St. John's National Academy of Health Sciences, Bangalore, India; Department of Chemical Engineering and Applied Chemistry, University of Toronto, Toronto, Canada; Hubert Department of Global Health, Rollins School of Public Health, Emory University, Atlanta, GA, USA; Department of Health Science and Technology, Laboratory of Human Nutrition, Institute of Food Nutrition and Health, ETH Zurich, Zurich, Switzerland

**Keywords:** fortification, salt, iodization, iodine, iron

## Abstract

*Could DFS help prevent iron deficiency and anemia?* Studies in controlled settings (efficacy) demonstrate that double-fortified salt (DFS; iron added to iodized salt) reduces the prevalence of anemia and iron deficiency anemia. Studies in program settings (effectiveness) are limited and reported differing levels of DFS coverage, resulting in mixed evidence of impact on anemia.

*What iron formulations are available and how do they affect iodized salt?* Ferrous sulfate and encapsulated ferrous fumarate (both with various enhancers and/or coating materials) are the main iron formulations currently in use for DFS. Adding iron to iodized salt may lead to adverse changes in the product, specifically discoloration and losses in iodine content. These changes are greatest when the iodized salt used in DFS production is of low quality (e.g., contain impurities, has high moisture, and is of large crystal size). DFS requires iodized salt of the highest quality and a high-quality iron formulation in order to minimize adverse sensory changes and iodine losses. Appropriate packaging of iodized salt is also important to prevent losses.

*What is known about the minimum requirements to manufacture DFS?* DFS producers must use high-quality refined iodized salt meeting the minimum standards for DFS production (which is higher than standards for salt intended for iodization alone), and an iron formulation for which there are rigid quality-assurance measures to ensure consistent quality and blending techniques. The actual proportion of iodized salt meeting the stringent requirements necessary for DFS production is unclear, but likely to be low in many countries, especially those with fragmented salt industries and a low proportion of industrially produced salt.

*What are the financial implications of adding iron to iodized salt?* As a result of higher input costs both for input salt and the iron compound, DFS is more expensive to produce than iodized salt and thus has a higher production cost. Various grades of iodized salt are produced and consumed in different sectors of the market. Experience in India indicates that, on average, producing DFS costs 31–40 US dollars/metric ton or 0.03–0.04 US dollars/kg more than high-quality refined iodized salt. The exact impact of this production-level cost difference on profit margins and consumer price is specific to the conditions of different salt markets. Factors such as transport costs, customary wholesale and retail mark-ups, and taxes all vary greatly and need to be assessed on a case by case basis.

*Is DFS in alignment with salt-reduction efforts?* The WHO has long recognized that salt iodization is an important public health intervention to achieve optimal iodine nutrition and is compatible with salt-reduction goals. Fortification of salt (with any nutrient) should not be used to justify or encourage an increase in salt intake to the public. Any effort to expand salt fortification to other nutrients should be done in close consultation with WHO and those working on salt reduction.

*What has been the experience with DFS delivery under different platforms?* To date, DFS has been introduced into the retail market and in social safety net (primarily in India) programs, but sensory changes in DFS have been raised as concerns. The higher price for DFS has limited expansion in the retail market. In social safety net programs where the cost of DFS is subsidized for beneficiaries, programs must consider long-term resourcing for sustainability.

*Overall*: The optimal production and delivery of DFS are still under development, as many challenges need to be overcome. There is a beneficial impact on hemoglobin in efficacy trials. Thus, if those conditions can be replicated in programs or the technology can be adapted to better fit current production and delivery realities, DFS may provide an effective contribution in countries that need additional food-fortification vehicles to improve iron intake.

 

## Introduction

Salt is a condiment that is consumed by almost all population groups in all countries, with little seasonal variation in consumption patterns and a relatively narrow range of consumption (1). Its chemical characteristics have made it an ideal vehicle to provide additional iodine to the diet without producing sensory changes. Salt iodization has been recommended by the WHO (2) and achieved remarkable success in the reduction in iodine deficiency disorders (3) and currently reaches 88% of households in low- and middle-income countries (4). With no doubt, it is one of the most successful public health interventions and has been credited with the virtual elimination of iodine deficiency disorders in most countries of the world.

This success has encouraged innovators and public health practitioners to explore the potential of iodized salt to carry additional nutrients to solve critical vitamin and mineral deficiencies, in particular iron, to address anemia caused by iron deficiency. Anemia has substantial physical, cognitive, and developmental consequences and affects 1.62 billion people globally (5, 6). Leveraging the success of salt iodization, there has been interest in exploring the feasibility of adding iron to iodized salt to produce double-fortified salt (DFS). The intent of DFS is to provide additional iron in settings where iron deficiency is widespread and contributes to an increased risk of anemia.

This brief outlines several key considerations for policymakers that should be in place for the successful implementation of iodized salt fortified with iron, without compromising the success of universal salt iodization (USI). These considerations are the result of a Global DFS Consultation convened by the Iodine Global Network (IGN) (7) to critically analyze all available information about DFS to define opportunities, risks, and challenges related to this new technology. The consultation included the development of a series of technical background papers (8–11) and was guided by a steering committee made up of international experts in and representatives of nutrition science, epidemiology, food technology, fortification programs, salt industry, and food industry who endorse this brief.

The brief presents the current evidence and experiences with DFS from these background papers as a series of critical questions that country policymakers and their advisors could consider regarding the potential viability of DFS in their national contexts.

## Could DFS Help Prevent Iron Deficiency and Iron Deficiency Anemia?

### What is the potential biological impact of DFS?

**FIGURE 1:**
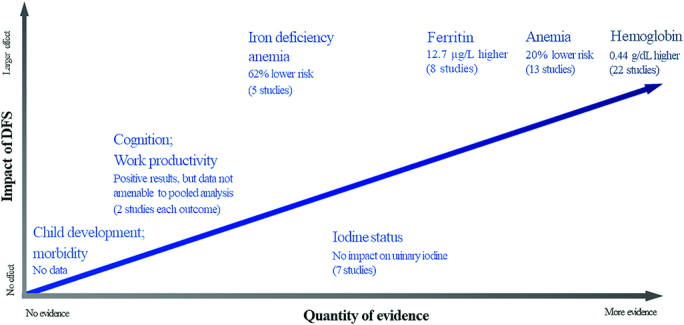
Available data from efficacy and effectiveness studies on the health impact of DFS. DFS, double-fortified salt. Reproduced from reference 8 with permission.

The efficacy of different DFS formulations has been tested in multiple studies. These formulations have included different iron compounds, including ferrous fumarate, ferrous sulfate, and ferric pyrophosphate, which are added to iodized salt. A systematic review and meta-analysis compared the effects of DFS versus iodized salt in women and children (8). It included 22 studies (16 from India, 2 from Morocco, 2 from Ghana, 1 each from Sri Lanka and Cȏte d'Ivoire) with a total of 52,758 participants. The key findings are presented in **Figure 1** and can be summarized as follows:

- Efficacy studies indicate a significant overall positive effect on measurements of biological status (i.e., on hemoglobin concentration and ferritin) and a reduction in the risk of anemia and iron deficiency anemia. There is insufficient evidence on the effects of DFS on functional outcomes, including cognition, development, and infections.
- Most iron compounds are efficacious but ferrous sulfate with sodium hexametaphosphate (SHMP), encapsulated ferrous fumarate, and micronized ferric pyrophosphate has the largest body of evidence.
- Overall, there was no significant difference in urinary iodine concentrations (UICs) comparing DFS with iodized salt, although 2 studies reported lower UICs with DFS, possibly due to losses of iodine in DFS during the storage process.
- For this analysis, 6 of 22 studies were rated as moderate or high quality, according to a published grading metric used by the authors. All 22 reported hemoglobin as an outcome; the 6 moderate- or high-quality studies reported stronger effects on hematological outcomes.

The impact of DFS seems to be greatest in populations with a high prevalence of anemia and when DFS provides ≥10 mg/d of iron per capita (8). In India, DFS is formulated to provide 100% of daily iodine requirements and 8.5–11 mg iron/d (∼30% of daily iron requirements for the targeted population) through an estimated 10 g of salt consumption per day (12). However, when formulated for the general population, DFS is unlikely to provide these nutrient amounts to children aged 6–24 mo because they consume less salt, as well as populations with low salt intake.

There is limited evidence demonstrating the effectiveness of DFS on health outcomes when implemented in program settings. There are 2 published effectiveness studies from India; however, neither study measured iron outcomes (only hemoglobin). Both studies experienced issues with project implementation, which may have affected potential impact on anemia. In one study, DFS was added to school meals and reported positive effects on hemoglobin but not on school performance or attendance (13). The second study was implemented through a social safety net platform, achieved low coverage, and had no significant effects on any of the studied outcomes (14). Effectiveness studies that focus on any potential bottlenecks in production, population coverage, or compliance are particularly important as they can help inform how DFS can be best implemented. [A large-scale study was undertaken in 2019 in Uttar Pradesh (15), India, of DFS effectiveness delivered through the public distribution system. The methodological details and results of this study were yet to be published at the time of the Global DFS Consultation.]

There is limited information on the potential negative health effects of DFS—for example, its potential to aggravate infections such as malaria, cause gut dysbiosis, and/or contribute to dietary iron overload. Risk of increased infections has not been observed with the lower amount of iron typically provided through fortified foods, but the higher amount of iron usually provided in supplement form has been associated with increased infections in some settings (16). Since iron added to other food vehicles (e.g., wheat flour, maize flour, rice, milk) has not led to adverse health effects (17–20), the assumption is that similar amounts of iron provided in DFS is also safe.

### Is DFS needed when other staple foods are being fortified with iron?

Any iron-fortification strategy should be developed using recent data available on dietary intake of iron at the population level (including from fortified foods and supplements), current iron status, and an understanding of the proportion of anemia that is caused by iron deficiency (21). From an efficacy point of view, wheat and maize flours, rice, and iodized salt fortified with iron in large industrial-scale facilities all have a similar potential to increase iron intakes and decrease the burden of iron deficiency in a population. However, selecting a food(s) to fortify should be based on coverage of the food across the population and per capita consumption of the food that is to be fortified, particularly among the neediest groups and fortification feasibility (structure and manufacturing capacity of the industry). After a food has been selected, compliance with legislation and the incremental cost of the fortified products influence a program's success (21).

DFS could add value when there is evidence that iron needs in the population are not being met through the diet, fortified foods, or other iron interventions. There are no comparative analyses of the cost-effectiveness of DFS relative to fortifying other foods. Additional factors also (discussed below) are also important considerations to the inclusion of DFS in a country's fortification program.

### Summary of the health impact of DFS

Efficacy studies (in controlled settings) indicate that DFS improves hemoglobin and ferritin concentrations and reduces the prevalence of anemia and iron deficiency anemia. However, there is no evidence of impact on anemia or iron deficiency from effectiveness studies (studies in program settings) (8), and no cost-effectiveness analyses of DFS have been done (22).

## What Iron Formulations Are Available and How Do They Affect Iodized Salt?

### Which iron formulations have been tested for addition to iodized salt?

Several iron formulations have been developed and evaluated, with the aim of increasing iron intake and avoiding adverse sensory changes or iodine losses when added to iodized salt (23–33). The most common of these iron formulations were classified as DFS 1 through 5 in 2014 (22) and have been updated as part of this review (**Table 1**).

**TABLE 1:** DFS Types 1–5^1^

	Type 1						
Characteristic	a	b	c	Type 2	Type 3	Type 4	Type 5
Iron compound	Ferrous fumarate			Ferrous sulfate heptahydrate	Ferrous sulfate monohydrate	Ferrous sulfate hydrate	Ferric pyrophosphate
Iron encapsulated?	No	Yes	Yes	No	No	Yes	No (micronized)
Iron encapsulates and/or additives	Soy stearine, titanium dioxide, HPMC, SHMP			SHMP	Malic acid, SHMP, sodium dihydrogen phosphate	Partially hydrogenated vegetable oil	N/A
Agglomeration method (if encapsulated)	N/A	Fluidized bed	Extrusion	N/A	N/A	—	N/A
Iodine encapsulated?	No			No	Yes	No	No
Iodine encapsulates and/or additives	N/A			N/A	Sodium bicarbonate, cellulose acetate phthalate, silicone	N/A	N/A

^1^Adapted from reference 22 with permission. DFS, double-fortified salt; HPMC, hydroxy-propyl methyl cellulose; N/A, not applicable; SHMP, sodium hexametaphosphate.

Due to studies reporting yellowing (29), iodine losses in humid conditions (30), or for proprietary reasons (28), of these, only encapsulated ferrous fumarate (DFS 1c, and before it, DFS 1b) and ferrous sulfate with SHMP (DFS 2) have been adopted for large-scale implementation in India, delivered as part of social safety net programs (10).

### What is the interaction between iron and iodine?

**FIGURE 2:**
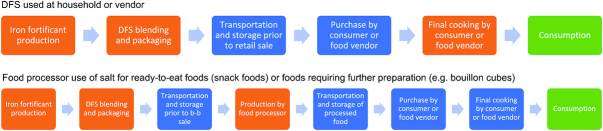
Two potential product and distribution pathways for double-fortified salt (DFS). When evaluating opportunity for a new food vehicle or the addition of a new nutrient to an existing food vehicle, research can be necessary to consider any potential for nutrient interactions, nutrient losses, or sensory changes (e.g., changes in color, taste, smell) at various points in a product’s pathway. Using DFS as an example (depending on the iron formulation or compound used), these could occur at production or processing steps (in orange) or storage and transportation (in blue). Whether these changes significantly affect the product from an iodine retention or consumer acceptance standpoint are realized at the consumption stage (in green). b-b, business-to-business.

Iron forms that are more easily absorbable by the body (ferrous forms) are highly reactive with iodine, through catalyzing the oxidation of iodate or iodide to iodine gas (34). Therefore, adding these iron forms to iodized salt can potentially lead to sensory changes and/or iodine loss (35). As a result, the successful production of DFS requires the separation of iron and iodine, which has been done through the encapsulation of iron, or use of a nonreactive iron compound such as ferric pyrophosphate, which is highly water insoluble. The quality of the input iodized salt (purity and moisture content), the iron formulation (e.g., quality of the iron encapsulation), and the quality of the production process all determine whether a high-quality DFS product can be manufactured that will not cause any adverse sensory or nutrient changes. These changes can occur at various stages along the supply chain between production and consumption—for example, blending and packaging, transportation through distribution networks, and to when it reaches consumers and is used in food preparation (23, 25–32), as highlighted in **Figure 2** (35). It is important to state that this evidence is limited by the fact that different methodologies have been applied across studies of the various DFS formulations.

### What has been the experience of adding iron to iodized salt?

Most formulations currently in use result in black spots (Type 1b/1c) or yellow discoloration (Types 2 and 5) in the DFS product (9). However, preventing moisture in salt (through the use of higher-quality salt refining and appropriate packaging and storage) may prevent DFS from developing black spots or turning yellow. The type of iron formulation and quality of input salt used affect the sensory changes of DFS. That is, salt of high purity (≥98% NaCl for Type 1b/1c and ≥99% for Type 2) and low moisture content (≤1.5%) is required for the manufacture of DFS in order to minimize sensory changes. However, even use of the highest-quality salt may not be able to eliminate sensory changes if the iron formulation used in DFS is of poor quality. Encapsulated formulations can minimize sensory changes, but the integrity of the encapsulation process is important and can be affected by poor quality assurance during production of the iron formulation (9).

### Iron formulation characteristics

Although there have been many iron formulations developed and tested for DFS, there are only 2 formulations that have been used in large-scale program settings:

DFS Type 1b/1c (33): Encapsulated ferrous fumarate developed by the University of Toronto with financial assistance from The Micronutrient Initiative (now Nutrition International). Type 1c uses a more sophisticated encapsulation method than Type 1b. DFS Type 1c consists of ferrous fumarate extruded with a binding agent, semolina. The extrusions are then cut into pellets. The size of these pellets is important and needs to closely match the crystal size of the salt to ensure even mixing and avoid segregation during any settling that occurs during transportation and distribution. The pellets are coated with titanium dioxide to mask and stabilize the color of the iron in the pellets before final encapsulation in soy stearate with additional titanium dioxide and hydroxypropyl methylcellulose. The encapsulated iron is added to the iodized salt at a rate of 0.50–0.57%.
  Because of its complexity, the encapsulated ferrous fumarate formulation is typically purchased from certified encapsulated ferrous fumarate manufacturers. If a country plans to produce DFS Type 1b/1c and prefers to source the encapsulated ferrous fumarate domestically (such as in India), a separate manufacturing plant, equipped with extruders, dryers, and coating equipment is necessary.
  Sensory changes that have been reported with the use of DFS Type 1b/1c include visible black spots (small iron particles) after production—hypothesized due to poor quality coating. There are also reports of darker food when DFS Type 1b/1c is used in cooking.
DFS Type 2 (36): Ferrous sulfate blended with stabilizing agents, developed by the National Institute of Nutrition (NIN) in India. This iron formulation uses ferrous sulfate heptahydrate as an iron source with SHMP added as a color stabilizer. These are added to the salt at the rate of 0.5% and 1%, respectively.
  Unlike DFS Type 1b/1c, ferrous sulfate and SHMP are both widely used in the food industry and can be purchased in the open market. Sensory changes related to DFS Type 2 include yellowing of the salt after production.

In particular, DFS Type 1b has been subjected to considerably more testing than other formulations to assess stability, bioavailability, efficacy, and consumer acceptance. Iron formulations for use in DFS continue to evolve. There are ongoing efforts to improve formulations using different iron compounds, enhancing agents, and other ingredients with the aim to further improve sensory characteristics, bioavailability, and nutrient stability.

The blending technique used during DFS production (which combines the iron formulation with iodized salt) can affect the integrity of the encapsulation (due to high heat), possibly leading to adverse sensory changes in the final product. As such, rigorous quality-control measures are needed for the both the iron formulation used in DFS and of the final DFS product (9).

Depending on the producer, DFS is typically packaged in low-density polyethylene co-extruded with polyethylene of varying sizes. The requirements for packaging DFS in order to maintain product stability are less clear given the limited production globally, but there is evidence that packaging material containing antioxidants can increase iodine losses (37). In India, it is advised that a nonlaminated polymer blend be used for DFS packaging (9). Adequate storage conditions of both loose and packaged DFS are needed to ensure stability and limit sensory changes. When minimum conditions are met, DFS is generally considered stable for at least 6 mo.

#### Producer perceptions

Producers who have considered DFS for the retail market noted concerns about sensory changes and potential market acceptability. Producers in India social safety net programs reported concerns about sensory changes and how they may affect consumer acceptance (9).

#### Consumer perceptions

Consumers have consistently reported concerns about black spots and/or color changes in the DFS product (10). Sensory changes result in uneven consumer acceptance, even where the price of DFS is subsidized by the Indian government in social safety net programs (38, 39). Experience from studies and programs in India, Argentina, Sri Lanka, and Nigeria shows that color change of the stored salt had an impact on consumer acceptance and uptake. No program reviewed reported changes in taste or smell when using DFS. Results of studies testing the acceptability of foods cooked with DFS are mixed (23, 27).

### Summary of iron formulations and effects of adding iron to iodized salt

There are basic challenges with combining iron with iodized salt, which may lead to adverse changes in the product in terms of color and nutrient stability. It is critical to mitigate the appearance of black spots or color changes in salt, minimize loss of iodine, and preserve the quality of the DFS throughout the supply chain. Not doing so might reduce iodine intake through salt, hindering the progress made to date in USI.

## What Is Known about the Minimum Requirements to Manufacture DFS?

### What are the technical requirements of producing DFS?

**FIGURE 3:**
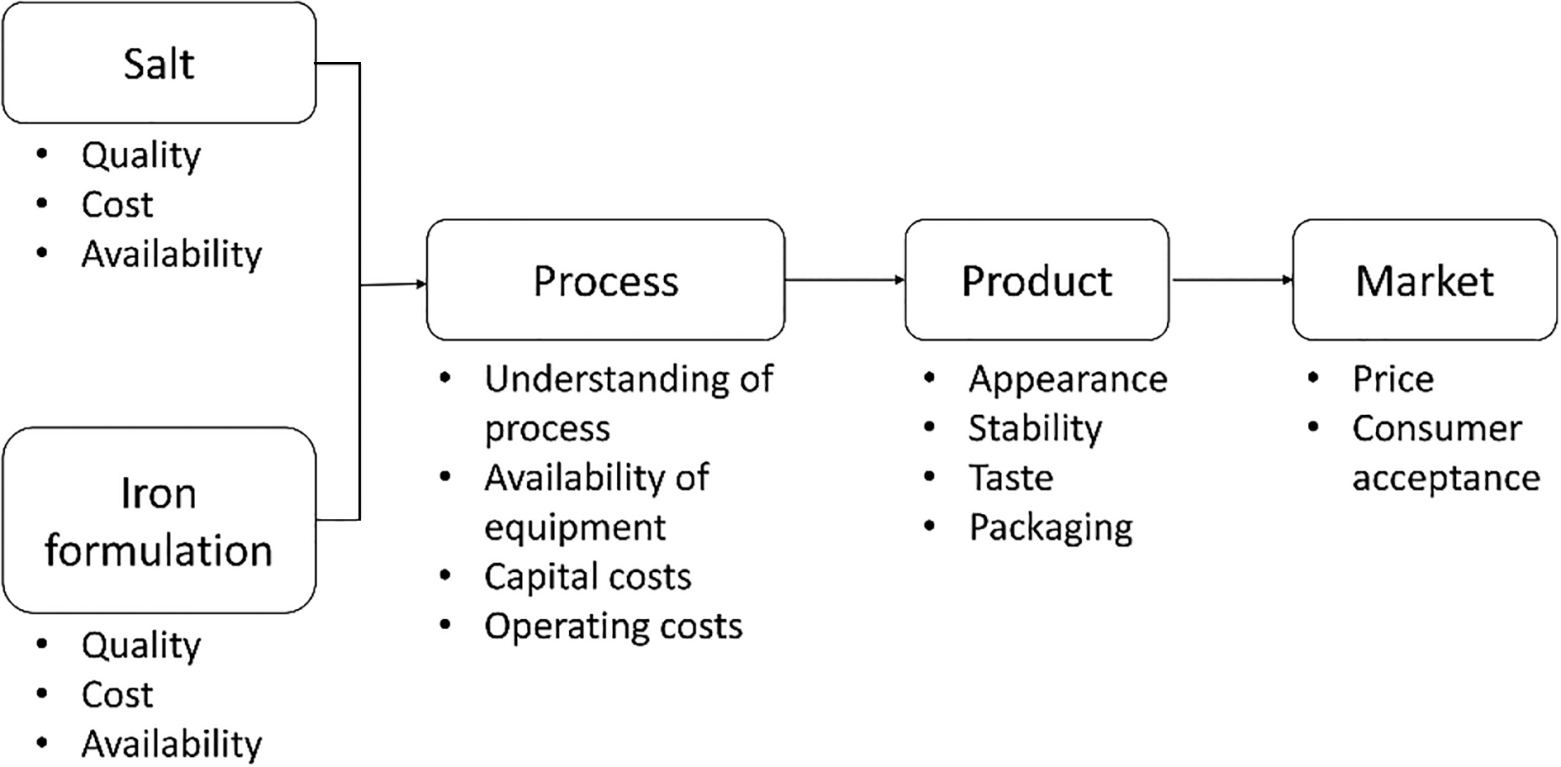
Necessary inputs for the production of DFS. DFS, double-fortified salt. Reproduced from reference 9 with permission.

The production of DFS is based on the addition of an iron compound to iodized salt. **Figure 3** illustrates the different inputs that are necessary.

#### Salt quality

A minimum requirement for DFS production is the availability of refined salt (vacuum crystallized or hydro-milled) for fortification that meets the required quality standard. **Table 2** provides examples of quality standards for input salt used in DFS production in India, for iodized salt in the East Africa region (40) (a typical iodized salt standard), and for the final DFS product in India (41). Of note, input salt for DFS production in India must be of greater NaCl content and lower moisture content than iodized salt in the East Africa region. Considering higher-quality standards for salt used for DFS, there is need for uniform global recommendations for salt used for DFS and for countries to ensure that their refined salt supply and specifications match these requirements.

**TABLE 2:** A comparison of salt standards when used in iodization or the production of DFS^1^

	Input salt for the production of DFS		Final product				
	IS 16,232:2014 Indian standard for DFS (19)		East African standard 35:2011 for iodized salt (20)			IS 16,232:2014 Indian standard for DFS (19)	
Characteristics	FS	EFF	Coarse salt	Crushed salt	Table salt	Fortified with EFF	Fortified with FS
Chloride content as (NaCl), % on dry matter basis, min	≥99	≥98	≥96.0	≥96.0	≥97.0	≥97	
Moisture content, drying at 105°C, %, m/m, max	≤1.5	≤1.5	≤4	≤4	≤3	≤1.5	
Matter insoluble in water, %, on dry matter basis, max	≤1.0	≤1.0	≤1	≤1	≤0.2	≤1.0	
Magnesium (Mg) water-soluble, % on dry matter basis, max	≤0.1	≤0.1	≤0.5	≤0.5	≤0.1	≤0.1	
Sulfate (as SO_4_), % on dry matter basis, max	≤1.1	≤1.1	≤0.5	≤0.5	≤0.50	≤1.1	
Acid-insoluble matter % m/m, max	≤0.3	≤0.3	—	—	≤0.2	—	
pH of solution, 20 g in 100 mL distilled water, using standard laboratory pH meter	3.5–5.5	3.5–7.5	7.0–8.0	7.0–8.0	7.0–8.0	3.5–7.5	3.5–5.5

^1^Reproduced from reference 9 with permission. DFS, double-fortified salt; EFF, encapsulated ferrous fumarate (used in DFS Type 1b and 1c); FS, ferrous sulfate; max, maximum; min, minimum; m/m, mass fraction.

#### Iron formulation characteristics

Access to an iron formulation that can be homogenously blended with iodized salt, with high-quality encapsulation (if relevant) and color masking (if relevant) to avoid iron and iodine interactions leading to iodine losses and/or discoloration, is needed.

#### Production process

An existing salt iodization plant can be modified to produce DFS by installing blending capacity for the iron formulation (9). For Type 2 and Type 1b/1c DFS, a 2-step blending process is necessary to ensure even mixing of the iron formulation and iodized salt. If producing Type 2 DFS (using ferrous sulfate and SHMP), first a concentrated mix of iodized salt with ferrous sulfate and SHMP is produced. In a second step the concentrated mix is blended with iodized salt and dried. If producing Type 1b/1c DFS (using encapsulated ferrous fumarate), a concentrated mix of encapsulated ferrous fumarate and iodized salt (at a 1:10 ratio) is produced using a small ribbon blender or screw mixer; slow blending is necessary to avoid disintegration of the encapsulated ferrous fumarate coating. The concentrated mix is then added to the rest of the bulk iodized salt and mixed to form the final DFS product, using larger ribbon blenders or screw mixers. The type of blending equipment required for DFS is widely used in the food industry and can be incorporated into an existing large-scale industrial salt-processing plant that already iodizes its salt. However, the blending process may affect the integrity of the encapsulated formulations via high temperatures and through abrasion. While the former can be managed by avoiding temperatures higher than 60°C, the effects of the abrasion are more difficult to manage and the equipment should be designed with careful consideration of the shear forces generated during mixing.

### What are the financial implications of adding iron to iodized salt?

#### For the producer

##### Initial capital investment

Where no or inadequate capacity to produce iodized salt of a suitable quality for DFS production exists, an initial investment will be required to establish a facility to process raw salt, or to improve existing refining capacity. The initial investment for a new processing plant varies between regions and it is hard to generalize costs; an example cost to install a new plant with 7200 tonnes/y capacity is US dollars (USD) 820,000 (9). However, if an industrial-scale factory is already in place and produces high-quality iodized salt, then investment costs to scale-up for DFS are limited to the incremental cost of installing blending and material handling equipment. Such incremental costs to produce DFS are estimated to be USD 65,000 for a 24,000 tonnes/y plant; if a 2-step blending process is required, there is an additional USD 3000 for the second blender (9).

##### Operating costs

Current experience indicates that the incremental cost of manufacturing DFS is from 31 USD/metric ton (MT) (0.03 USD/kg) for Type 2 DFS to 40 USD/MT (0.04 USD/kg) for Type 1b/1c DFS, over and above the cost of producing high-quality refined iodized salt (9). These marginal costs could be higher if an iodized salt producer requires adding processing steps to produce salt that meets the DFS standard.

#### For the government

The cost of DFS to the government will depend on the delivery platform. India is, by far, the largest producer and user of DFS, distributing DFS at a subsidized price through various social safety net programs (10). The total cost of these subsidies to the Government of India has not been reported. Monitoring costs for regulatory agencies will include testing to ensure quality of the iron formulation and the final DFS product, which may require additional laboratory equipment and training. The cost-effectiveness of DFS is unknown.

#### For the consumer

With regard to price increases, to date, DFS entry into the retail market in India has been minimal; producers in India and several other countries where DFS has been considered have reported concerns about cost and consumer acceptance (9).

### How do you monitor the addition of iron to iodized salt (quality of the iron formulation as well as the DFS product)?

A robust monitoring system is required that *1*) ensures input iodized salt meets required standard/purchase specifications, as this will help limit sensory changes and iodine losses, and *2*) ensures that the iron formulation meets the required chemical composition, including coating integrity requirements (if an encapsulated iron formulation is used). As above, this may require investments to develop the necessary testing capacity.

After production, the DFS product can be tested for iron and iodine content, color changes, and coating integrity (in the case of Type 1b/1c DFS). Ensuring nutrient content will improve the likelihood of DFS's impact on iron deficiency and reduce any risks to the USI program due to iodine losses.

### Summary of production requirements for DFS

Producers should use input iodized salt meeting the minimum DFS standard and an iron formulation for which there are rigid quality-assurance measures to ensure consistent quality. After the initial setup, adding iron to iodized salt increases the production cost of fortified salt by 31–40 USD/MT or 0.03–0.04 USD/kg.

## Is DFS in Alignment with Salt-Reduction Efforts?

It is important that any salt-fortification program be aligned with public health goals to reduce salt consumption in the population to recommended amounts. This is due to concerns that high salt intake may increase the risk of hypertension and other noncommunicable diseases (42). In fact, there is a close synergy between these 2 public health strategies, and it is important that communication and messages are developed to reinforce their complementarity. To this end, the IGN, WHO, and other partners have outlined a number of basic principles and areas for collaboration to reinforce the close alignment between salt iodization and salt reduction. These were summarized during a WHO Expert Consultation, 21–22 March 2007, Luxembourg, further reinforced as part of an interagency meeting in Sydney, Australia, in 2013 (43) and include the following:

- Policies for salt iodization and reduction in salt intake to <5 g/d are both necessary and compatible.
- USI is the recommended strategy to control iodine deficiency, and successful programs should continue and be sustained.
- Reliance of salt as a vehicle for iodine should not be used to justify, promote, or otherwise cause an increase in salt intake to the public and additional vehicles for iodine should be explored.
- WHO guidelines for the fortification of salt with iodine recommends iodine addition at 20 mg/kg based on an average salt intake of 10 g/d at the population level (44). If average intake of salt drops below 10 g/d, iodine addition levels may be raised to maintain iodine intake.
- Changes in population salt intakes and iodine status need to be assessed over time via monitoring of urinary sodium and urinary iodine. These data, combined with data on the different sources of salt and iodine in the diet, can be used to adjust iodine amounts in salt fortification accordingly to ensure optimization of these 2 public health strategies.

### Summary of DFS in the context of salt-reduction recommendations

As salt intakes decrease in a population (through salt reduction efforts or otherwise), it is possible to adjust iodine fortification amounts in salt to ensure sufficient iodine at any given salt intake. The same principles should guide the addition of other nutrients such as iron. Any effort to add nutrients to salt beyond iodine should be done in close consultation with WHO and those working on salt reduction to align messages and undertake complementary monitoring to track progress.

## What Has Been the Experience with DFS Delivery under Different Platforms?

### What delivery platforms have been used to deliver DFS?

Several countries have piloted and/or studied DFS in different contexts, including Côte d'Ivoire, Kenya, Morocco, Nigeria, Philippines, and Sri Lanka. India has used several large social safety net programs that have provided DFS to >70 million beneficiaries (10). In addition, DFS has been produced on a voluntary basis for the open market in India, and on a very limited scale in Argentina. There are no countries with mandatory requirements to fortify salt with both iron and iodine. In India, 2 social safety net programs [Midday-Meal (MDM) program and Integrated Child Development Services (ICDS)] have policies that require the procurement of DFS.

### What have been the implementation experiences?

#### Social safety net programs

The most programmatic experience has been in India, where DFS has been distributed via different social safety net programs, including MDM, ICDS, and most notably, through the Public Distribution System (PDS) in the states of Bihar, Andhra Pradesh, Tamil Nadu, Madhya Pradesh, Uttar Pradesh, and Gujarat. Under these programs, DFS has been provided for free or at a subsidized price (10). Challenges related to providing DFS through India's social safety net programs revealed were as follows:

- Perceptions of DFS as an inferior product;
- Inadequate or opaque state-level procurement process, leading to DFS quality issues, most notably color changes;
- Poor or inadequate communication to consumers regarding DFS and expectations regarding any potential color changes due to the addition of iron; and
- Program difficulties ensuring DFS quality for beneficiaries. Although a DFS standard exists (for the finished product), there are no standards for the iron formulations added to DFS or regulatory monitoring protocols to assess and ensure quality of DFS.

In India, development partners and donors have successfully advocated for the inclusion of DFS in social safety net programs. At a policy level, the Government of India has supported the widespread production and consumption of DFS. Where salt is included as a basic commodity in the PDS program, and in MDM/ICDS programs, states have been directed to use DFS (45–47). In these state programs, the cost of DFS has been subsidized at a price that is lower than the market price for iodized salt. Given the financial implications, it is ultimately a decision of individual state governments to decide whether they adopt DFS in their social safety net programs.

#### Retail market

Currently, there is no large-scale use of DFS in retail settings, except for a few producers in India and a very small market in Argentina. In Argentina, the price premium is 50–60% as compared to iodized salt and is therefore targeted to higher-end consumers (10). There have been previous efforts in Kenya and Nigeria to introduce DFS in the retail market with the assistance of development agencies, but these were discontinued because of low sales, adverse color changes in the salt, and concerns that the higher price would not be acceptable to potential consumers without subsidies.

### Can DFS follow the same delivery platform as iodized salt?

Globally, it is mandatory for iodine to be added to edible salt (through processed foods or household use) in 128 countries (48), which has been one of the key factors that has made USI programs successful. Where there is a high level of compliance by the salt industry, mandating iodization has led to a significant increase in the supply of iodized salt, increased iodine intakes in populations, and a reduction in the burden of iodine deficiency. Even with mandatory legislation in place, there has still been a need for monitoring at all levels of the salt market to ensure compliance with the relevant standards and regulations.

Initiating mandatory salt iodization has been possible for several reasons: iodine added to salt causes minimal sensory changes, even in salt of low quality; there are simple tests available to measure the presence of iodine in iodized salt; there is an extensive, stable supply and procurement system for iodine premix; and the cost of adding iodine is a modest cost to salt production and this additional cost can be passed on with only limited resistance from the consumer.

Unlike iodized salt, current formulations of DFS have sensory, quality, and cost considerations that could be challenging. Production of DFS requires high-quality, refined salt that may not be within the production capacity of medium- and small-scale salt producers. The final DFS product and foods cooked with DFS may experience color changes. Experiences with Type 1b/1c have shown that the same food prepared with DFS may be a darker color than when iodized salt is used (39).

These operational realities present challenges to making DFS mandatory in the retail market. However, these constraints may have less of an effect in government social safety net programs, where DFS supply chains can potentially be better controlled and the cost of DFS may be subsidized.

### Summary of implementation experiences

India's extensive social welfare programs have provided a unique opportunity to distribute DFS at scale, with strong support from development partners and some state governments. Social welfare programs require committed state governments to subsidize the product, ensure effective procurement and delivery systems, and monitor the use of high-quality DFS formulations and iodized salt to mitigate against color changes. In comparison, there has been minimal expansion of DFS into the retail market under mandatory or voluntary settings, providing limited experience with this platform.

## Research Needs

DFS technology and program implementation is an evolving field and there is a critical need to explore the following in order to *1*) improve the quality of DFS in use and *2*) understand its implications and integration in public health programs:

- Develop global quality standards for DFS and the iron formulation(s) used in DFS.
- Undertake additional research to identify efficacious iron formulations for use in DFS that do not produce significant sensory changes or cause iodine losses.
- Explore technological options for DFS to be manufactured with lower-quality input salt (e.g., lower purity and higher moisture), while maintaining acceptable sensory qualities and iodine retention.
- Review and, if necessary, further test iodine stability in DFS formulations under real-world production conditions.
- Conduct cost-effectiveness analyses of different DFS formulations and of DFS in comparison with other fortification interventions for the prevention of iron deficiency and iron deficiency anemia.
- Evaluate consumer sensitivity to the price of DFS (and willingness to pay) to develop guidance on production cost levels likely to result a product acceptable in the open market.
- In countries interested in DFS, assess domestic capacity to produce DFS quality salt and monitor DFS stability throughout the supply chain; if a consumer-led market for DFS is considered, an important factor is an acceptable price to both consumers and producers.

## Notes

This consultation was made possible through funding support from UNICEF (through a grant from USAID) and the Bill & Melinda Gates Foundation (OPP1195090A).

Author disclosures: Greg S Garrett/GSG was formerly a staff member at the Global Alliance for Improved Nutrition (GAIN) and ThinkWell. His contributions to the DFS Consultation began when he was a staff member at GAIN and were completed after he joined ThinkWell in November 2019. AVK is a member of the Journal's Editorial Board and played no role in the Journal's evaluation of the manuscript. RK is a UNICEF staff member. The opinions and statements in this brief are those of the author and may not reflect official UNICEF policies. The other authors report no conflicts of interest.

Publication costs for this supplement were defrayed in part by the payment of page charges. The opinions expressed in this publication are those of the authors and are not attributable to the sponsors or the publisher, Editor, or Editorial Board of *The Journal of Nutrition*. The Guest Editor for this supplement was Becky Tsang, Iodine Global Network, Seattle, WA, USA, and she has no conflicts of interest.

Address correspondence to Robin Houston (e-mail: houston.robin@gmail.com).

Abbreviations used: DFS, double-fortified salt; ICDS, Integrated Child Development Services; IGN, Iodine Global Network; MDM, Midday Meal; MT, metric ton; PDS, Public Distribution System; SHMP, sodium hexametaphosphate; UIC, urinary iodine concentration; USD, US dollars; USI, universal salt iodization.
